# Feedback From Facial Expressions Contribute to Slow Learning Rate in an Iowa Gambling Task

**DOI:** 10.3389/fpsyg.2021.684249

**Published:** 2021-08-09

**Authors:** Shushi Namba

**Affiliations:** Psychological Process Team, Guardian Robot Project, RIKEN, Kyoto, Japan

**Keywords:** facial expression, Iowa Gambling Task, social cognition, learning, decision making

## Abstract

Facial expressions of emotion can convey information about the world and disambiguate elements of the environment, thus providing direction to other people’s behavior. However, the functions of facial expressions from the perspective of learning patterns over time remain elusive. This study investigated how the feedback of facial expressions influences learning tasks in a context of ambiguity using the Iowa Gambling Task. The results revealed that the learning rate for facial expression feedback was slower in the middle of the learning period than it was for symbolic feedback. No difference was observed in deck selection or computational model parameters between the conditions, and no correlation was observed between task indicators and the results of depressive questionnaires.

## Introduction

Our daily interactions are often ambiguous, and facial expressions of emotion can help disambiguate social situations by providing social information (Van Kleef, 2009, 2017). According to the theory of affective pragmatics, an emotional expression can incorporate communicative moves, namely, the things we do as we express emotions, and communicative effects, namely, the things we do by expressing emotions in nonverbal modules (Scarantino, 2019). For example, we can consider that a facial expression of fear can lead others to believe in a warning of danger and lead them to engage in safer behavior (Reed and DeScioli, 2017a). Likewise, facial expressions of sadness elevate the credibility of a loss message, which can lead observers to seek to aid an expresser if the loss remains uncertain (Reed and DeScioli, 2017b). Duchenne smiling, which includes eye constriction, also increases the credibility of a speaker’s words for directing auditors’ actions (Reed et al., 2018). In other words, facial expressions related to emotional meaning can establish the credibility of certain facts and convey information that is capable of directing other people’s behavior.

Work investigating the functions of emotional expressions has focused on two expressions in particular: happiness and anger. Previous studies have indicated that facial expressions of happiness signal greater acceptance and induce affiliation in observers, while expressions of anger signal greater rejection and induce avoidance in observers (Kraut and Johnston, 1979; Gottman and Levenson, 2002; Fischer and Roseman, 2007; Stins et al., 2011; Heerdink et al., 2015; Namba et al., 2020; Perusquía-Hernández, 2020). However, little work has investigated the functions of facial expressions in relation to learning patterns over time. Lin et al. (2012) used reward-learning tasks using facial expression and monetary feedback and found that the anatomical substrates of the two overlapped, while learning performance was slightly slower in the context of feedback from facial expression. Thompson and Westwater (2017) found no difference between facial expression and monetary feedback in the performance of the Go/No-Go learning task that aimed at determining the ability of an individual to inhibit a response that is considered as an inappropriate and orthogonalized action and an outcome valence. Moreover, Case and Olino (2020) developed and used learning tasks that use reward/punishment feedback to investigate the difference between monetary and facial expression feedbacks; however, they were unable to ascertain the main effect of this difference.

There may be little or no difference in learning performance between social and monetary feedback; however, it remains elusive whether facial expression can contribute to the credibility of feedback for learning over time. In our daily lives, there are at least two types of feedback that use facial expressions: one type is the case where a facial expression itself is a reward/punishment and the other one is the case where a facial expression facilitates the function of a reward/punishment. Previous studies have dealt with the former case (e.g., Lin et al., 2012; Thompson and Westwater, 2017; Case and Olino, 2020), but none of the studies have investigated the latter case. For examples of facial expressions facilitating the reward/punishment feedback, children might receive rewards in the form of candy from their parents, and that reward might come with a smile. When the director of a department scolds a member of the department, he may also frown at the same time, resulting in an emphasis on the normative message being delivered. Children could perceive candy with a smile as a stronger reward, while a member of the department could perceive a rebuke with a frown as a stronger punishment. Facial expressions can affect the interpretation of verbal statements (Krumhuber and Manstead, 2009). Therefore, a facial expression can influence the function of feedback, such as a reward/punishment, and it is important to provide evidence regarding the function of facial expressions in feedback.

In relation to learning in decision-making situations, several laboratory studies have used the Iowa Gambling Task (IGT) to proxy real-life decision-making under conditions of ambiguity (Bechara et al., 1994). With IGT, participants are required to choose four decks that will receive feedback in the form of either a reward or punishment and aim to get the reward as much as possible. Some decks will tend to reward the player more often than other decks (advantageous decks and disadvantageous ones) and therefore the performance of a player can be computed based on the number of advantageous decks that participants select. The prevailing interpretation of IGT data has been that healthy participants first explore different decks and then exploit the most profitable deck. It has been assumed that the lack of somatic responses when selecting disadvantageous decks leads to various clinical and neurological problems (Bechara et al., 1994; Must et al., 2006; Agay et al., 2010).

However, Steingroever et al. (2013) analyzed eight IGT data sets (*N* = 479), and their findings revealed that healthy participants do not demonstrate a systematic decrease in the number of switches across trials. These findings led to another issue, which is whether components can work well in an IGT that approximates real-life reward learning under the conditions of ambiguity. The type of feedback appears to be one of the components that induce different IGT performances. Although previous studies that used several reward-learning tasks found little difference between facial expressions and monetary feedback, none investigated whether facial expressions can facilitate the function of reward/punishment in learning tasks. It is expected that learning can be promoted by adding feedback in the form of facial expressions in addition to the normal monetary feedback that is always provided.

The depressive symptom can also be related to IGT performances. Must et al. (2013) found that the performance of depressed persons on various decision-making tasks, including IGT, was impaired. More interestingly, Case and Olino (2020) found that in social IGT, which uses facial expression feedback instead of monetary feedback, participants in depressive symptoms played less from advantageous decks over time. Therefore, when exploring the feedback-facilitation effect of facial expressions, it is important to add a variable of depressive symptoms.

To gain further insight that is beyond the constraints of the rough interpretation of behavior, a computational approach would also work well. It is well known that computational models provide a means of decomposing performance and determining the parameters associated with fine-grained sources for behavioral patterns (Worthy et al., 2013). For instance, in the IGT, if a participant selects a disadvantageous deck, there may be several reasons for this: they may be insensitive to loss, they may have failed to learn the contingencies; they may be more inconsistent with their choices (Ahn et al., 2016). Chan et al. (2014) used computational modeling and found that the anorexia group in their study showed challenges to their learning or memory regarding their behavioral history. Ahn et al. (2014) also showed that heroin users displayed insensitivity to losses. Therefore, the computational model can be an informative approach to produce a finer-grained understanding of the performance of learning tasks.

In sum, this study aimed to investigate whether learning can be promoted by adding feedback in the form of facial expressions in addition to the normal monetary feedback given in IGT. To ascertain the effect of facial expression feedback, the researchers added a control condition that included feedback in the form of symbols (∘ and ×). In Japan, ∘ has been conventionally used as a feedback for positive or correct evaluation, while × has been used as a feedback for negative or incorrect evaluation. These two conditions have a common similarity—they provide information and monetary feedback. The difference between the two is the type of signal, that is, facial expressions or symbols. Additionally, this study also aims to confirm the differences in the effect of depressive symptoms regarding learning rate between the two feedback conditions. It also examines the behavioral indices using a computational approach and attempts to provide more detailed insight from the aforementioned results.

This study investigated the first hypothesis that facial expression feedback facilitates learning more than symbolic feedback. If many emotional expressions were selected as component parts of fully fledged adaptive action (Darwin, 1872), it can be predicted that social feedback (by means of facial expressions) contributes to the credibility of monetary feedback and promotes learning. The second hypothesis was that there is an interaction effect between feedback condition and depression on the learning rate of IGT. Therefore, a decrease in performance with increasing depression can be observed when feedback is in the facial expression condition. This hypothesis is consistent with Case and Olino (2020) findings. The final hypothesis is that the computational parameters of the behavioral data can provide fine-grained understanding of the results. To ascertain this, this study exploratively investigates the statistical model that fits and explains the data. However, the Outcome-Representation Learning model (ORL) model, which assumes that the expected value and win frequency for each deck are tracked separately, has been proposed as the best model at present (Haines et al., 2018). Therefore, the ORL model would fit the data better than other reinforcement learning models. There will be differences in the learning rate derived from the computational model between feedback conditions because facial expression feedback is expected to facilitate reward/punishment learning.

## Materials and Methods

### Participants

Data were collected from 57 undergraduate students (33 female, 24 male; *M*_*age*_ = 19.60, SD = 0.49, and range = 19–20). They participated on a voluntary basis. All participants were native Japanese speakers with normal or corrected-to-normal vision. Written informed consent was obtained from each participant before the study, in line with a protocol approved by the Ethical Committee of the Graduate School of Education, Hiroshima University. The sample size was chosen based on previous review of IGT using healthy participants (Steingroever et al., 2013). The average number of participants used in the 39 studies was approximately 37 (range = 10–141; see Table 2 in Steingroever et al., 2013), and the number of participants in this study was 1.5 times this average, which can be considered as sufficient.

### Iowa Gambling Task

**FIGURE 1:**
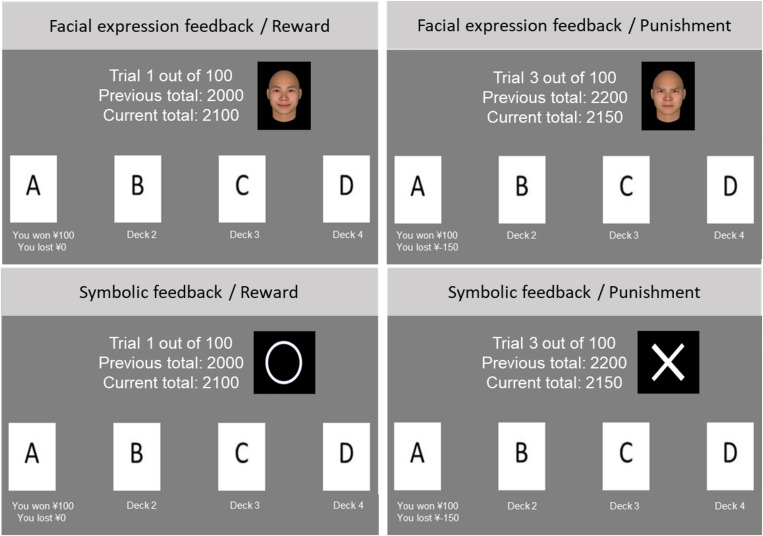
Example feedback condition in the Iowa Gambling Task. The upper panel shows social feedback, whereas the lower panel shows symbolic feedback.

This study used the standard computerized version of the IGT developed by PsychoPy2 (Peirce et al., 2019). Table 1 indicates the payoff of the IGT. In this task, participants were instructed to pick up one card from an array of four decks (A, B, C, and D), and were informed that their task was to maximize gain over 100 trials. As Table 1 indicates, the first two decks (A and B) could be considered disadvantageous, while the latter two decks (C and D) could be considered advantageous. The most appropriate choice for the participants was to avoid selecting from the disadvantageous deck and increase selection from the advantageous deck as far as possible through 100 trials. Using the standard IGT, this study added the feedbacks. When presenting feedback, one group obtained feedback in the form of facial expressions, and the other obtained feedback in the form of symbols. If the total amount of money that participants received was positive, a smile or a ∘ was presented, and if it was negative, an angry face or a × was presented. The avatar expressions were generated using FaceGen Software. These avatars were used with all parameters (e.g., gender and racial group) set to the average. Figure 1 shows an example of the experimental situations in the two conditions of IGT. For the purposes of transparency of the study and open science, all experimental codes were uploaded to OSF^1^.

**TABLE 1:** Payoff distribution of the Iowa Gambling Task.

Deck	A	B	C	D
Gain from each trial ($)	1.00	1.00	0.50	0.50
Loss amount(s) in each set of 10 trials	–1.50	–12.50	–0.25	–2.50
	–2.00		–0.50	
	–2.50		–0.50	
	–3.00		–0.50	
	–3.50		–0.75	

### Self-Report Questionnaire

This study applied two questionnaires, the Center for Epidemiological Studies Depression Scale (CES-D; Radloff, 1977) and the Short Intolerance of Uncertainty Scale (SIUS). The CES-D was developed to measure the degree of the depressive tendency, and the Japanese version was validated by Shima et al. (1985). This scale includes 20 items on a 4-point scale, ranging from 0 (rarely or not at all) to 3 (most or all of the time) over the time period of the previous week. The SIUS was developed to measure the tendency to perceive uncertainty as threatening, regardless of the true probability of the threat (Carleton et al., 2007). This scale consisted of 12 items presented on a 5-point scale, from 1 (not at all characteristic of me) to 5 (entirely characteristic of me). Takebayashi et al. (2012) also created the Japanese version and validated it. In this study, the average score of the items for the CES-D was 1.26 (SD = 0.54), and the average score of items for SIUS was 3.32 (SD = 0.65). The SIUS metrics were measured for another relevant research project on IGT and, the results were therefore not reported using this questionnaire.

### Procedures

After they had provided written informed consent, all participants performed the IGT. Participants were randomly assigned to one of two groups: feedback with facial expressions (*N* = 29, 16 female, 13 male; *M*_*age*_ = 19.52, and SD = 0.51) and feedback with symbols conditions (*N* = 28, 17 female, 11 male; *M*_*age*_ = 19.68, and SD = 0.48). The latter condition was regarded as the control condition. This assignment was performed in a random manner. The participants received the standard instruction for IGT, and not for the feedback conditions (facial expressions and symbols). Before performing the main IGT, the participants were asked to imagine or assume that the money they were set to receive was real money. Next, we assessed participants’ self-reported depressive tendency, using the Japanese version of CES-D (Cronbach’s α = 0.88) and the Japanese version of the SIUS (Cronbach’s α = 0.80) to assess the tendency to perceive uncertainty.

### Computational Model

This study tried to fit three models: the Prospect Valence Learning model with the delta rule (PVL-delta; Ahn et al., 2008), the Value-Plus-Perseverance model (VPP; Worthy et al., 2013), and the ORL (Haines et al., 2018). The PVL-delta model used a Rescorla–Wagner updating equation (Rescorla and Wagner, 1972) and provided four parameters. The learning-rate parameter (0 < *A* < 1) was used to weight recent outcomes for updating the expected value. The shape parameter (0 < α < 2) determined the shape of the utility function, and the loss aversion parameter (0 < λ < 10) represented the control of the effect of losses relative to gains. The high and low consistency parameters (0 < *c* < 5) represented more deterministic or more random choices. In addition to all of the parameters of PVL-delta, the VPP model included an additional four parameters. The perseverance decay parameter (0 < *k* < 1) indicates how much the perseverance strength of all decks is discounted on each trial. The perseverance gain (−∞ < ϵ_*p*_ < ∞) and loss (−∞ < ϵ_*n*_ < ∞) impact parameters show how the perseverance value changes after wins and losses, respectively. The reinforcement-learning weight parameter (0 < ω < 1) was weighted to the reinforcement learning versus the perseverance term. For the ORL model, two learning-rate parameters were used for reward (0 < *A*_*rew*_ < 1) and punishment (0 < *A*_*pun*_ < 1) outcomes. Both parameters were used to update expectations after reward and punishment outcomes. The ORL model was also used to describe win frequency for each deck, and the decay parameter (0 < K < 5) indicated how far players forgot their own deck selection. The frequency weight parameters (−∞ < β_*F*_ < ∞) showed the frequency of preference for a given deck, and the perseverance weight parameters (−∞ < β_*P*_ < ∞) controlled whether to switch or stay with recently chosen decks.

Prospect Valence Learning-delta is the simplest reinforcement learning model, and VPP is a PVL-delta model that includes a top-down strategy that is a win–stay lose–shift. ORL is presumed to be a model that includes an index of win frequency in addition to the reinforcement learning model. The order of complexity based on the number of parameters is PVL-delta (4), ORL (5), and VPP (8). The more complex the model, the better it fits and the more likely it is to over-fit the data. Because of its complexity, the parameters reflected in each model do not necessarily explain the same variance, even with the same name. Based on previous studies (e.g., Haines et al., 2018), ORL has been considered as the best model using comprehensive results, such as fitting, simulation, and parameter recovery.

The analyses were performed in R (3.6.1, R Core Team, 2019) using the hbayesDM package (Ahn et al., 2017). The details of the used models have been described online^2^. The model computation given above was set as the default in the hbayesDM package. The value of Rhat for all parameters equaled 1.0, indicating convergence across the four chains.

### Statistical Analysis

To compare the feedback conditions, we used the regression model dropping intercept covariance, where the number of advantageous deck selections every 20 trials were predicted variables, and the feedback condition and standardized CES-D score and their interactions were predictors. It had been expected that there would be an interaction effect between the number of trials and the feedback condition. More precisely, the number of advantageous deck selections would be facilitated in a facial expression feedback condition. Additionally, the current study evaluated the interaction between feedback, number of trials, and CES-D. According to Case and Olino (2020), depressive symptoms reduce the learning rate in the facial expression feedback.

Next, we checked the results of the computational model and confirmed model fit for each social and symbolic feedback condition by comparing widely applicable information criterion (WAIC; Watanabe, 2010). Following this, the parameters were compared according to the differences in the posterior distribution between conditions. Finally, we created a correlation matrix between the simple IGT indicators “frequencies of each deck,” measured depressive symptoms with questionnaires, and derived each parameter from computational models. All analyses were performed using R statistical software, version 3.6.1 (R Core Team, 2019), alongside the “brms,” “corrr,” and “tidyverse” packages (Bürkner, 2017; Wickham et al., 2019; Kuhn et al., 2020).

## Results

To check the effect of other variables, such as gender and age, we used the regression model dropping intercept covariance, where the number of advantageous deck selections for every 20 trials were predicted variables and the participants’ gender and standardized age were predictors. We controlled all *p*-values by a false discovery rate using the Benjamini–Hochberg procedure (Benjamini and Hochberg, 1995). Table 2 shows the results of this regression. Because the male participants showed more selection of advantageous decks than their female counterparts (*β* = 1.45, *t* = 2.05, and *p* = 0.06), the main analysis included gender predictors for the control variable. Additionally, the number of advantageous decks selected in the last 20 trials increased compared to the first 20 trials (*β* = 1.79, *t* = 3.30, and *p* = 0.003). Therefore, IGT learning can be interpreted as successful to some extent. As for this significant effect, the *post-hoc* sensitivity power analysis using the simr package (Green and MacLeod, 2016) indicated that this sample size was sufficient to detect a regression coefficient for the last 20 trials, with a significance level of α = 0.05 and 90% power.

**TABLE 2:** Estimated parameters using the regression model.

Parameter	Mean	*t* value	*p* value
0–20 (Intercept)	7.76	13.52	>0.001
21–40	1.04	1.91	0.07
41–60	1.83	3.36	>0.003
61–80	1.56	2.88	>0.008
81–100	1.79	3.30	>0.003
Age	–0.02	0.05	0.96
Gender	1.45	2.04	0.06

**Random effect**	**Variance**		

Participants		5.31	
Residual		8.39	
Conditional *R*^2^		0.43	

**FIGURE 2:**
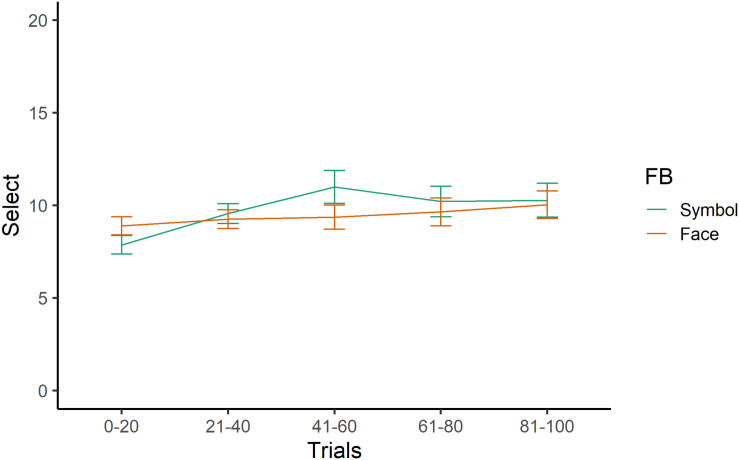
Learning curves for social and monetary feedback. Line plots represent means, and error bars represent standard errors. The *y*-axis indicates the number of advantageous decks that the participants selected.

Table 3 shows the main results using the regression model that included gender as a control variable. All the *p*-values were adjusted by the false discovery rate using the Benjamini–Hochberg procedure. We checked the differences in the feedback conditions. Compared to the first 0–20 trials, the learning rate under the face feedback condition was slower in the central 41–60 trials (Figure 2; β = −2.68, *t* = 2.46, and *p* = 0.05). For this effect, the *post-hoc* sensitivity power analysis indicated that this sample size was sufficient to detect a regression coefficient for the last 20 trials, with a significance level of α = 0.05 and 70% power. There were no effects for depression, condition, or their interactions.

**TABLE 3:** Estimated parameters using the regression model.

Parameter	Mean	*t* value	*p* value
0–20 (Intercept)	7.22	9.50	>0.001
21–40	1.69	2.22	0.08
41–60	3.14	4.12	>0.001
61–80	2.35	3.08	>0.001
81–100	2.41	3.17	>0.001
Feedback (FB)	1.12	1.14	0.31
CES-D	–0.28	0.62	0.54
Gender	1.44	2.00	0.12
21–40*FB	–1.34	1.23	0.31
41–60*FB	–2.68	2.46	0.05
61–80*FB	–1.60	1.47	0.27
81–100*FB	–1.27	1.17	0.31
0–20*FB*CES-D	1.36	1.47	0.27
21–40*FB*CES-D	0.69	0.75	0.51
41–60*FB*CES-D	0.65	0.71	0.51
61–80*FB*CES-D	1.19	1.28	0.31
81–100*FB*CES-D	1.31	1.42	0.27

**Random effect**	**Variance**		

Participants		5.27	
Residual		8.41	
Conditional *R*^2^		0.45	

A comparison of WAIC between computational models indicated that the ORL model is the best fit to the data (WAICs for social feedback: PVL-delta = 7,222, VPP = 6,613, and ORL = 6,475; WAICs for symbol feedback: PVL-delta = 7,435, VPP = 6,362, and ORL = 6,310). In the subsequent analysis, the parameters calculated by the ORL model were used. As for the facial expression feedback condition, the parameters were as follows: *A*_*rew*_ Mean [95% CI] = 0.15 [0.11, 0.20]; *A*_*pun*_ = 0.04 [0.03, 0.06]; *K* = 0.28 [0.13, 0.39]; β_*F*_ = 2.03 [1.40, 2.61]; and β_*P*_ = −2.35 [−3.37, −1.36]. For the symbolic feedback condition, the parameters were as follows: *A*_*rew*_ Mean [95% CI] = 0.19 [0.13, 0.26]; *A*_*pun*_ = 0.06 [0.04, 0.08]; *K* = 0.40 [0.26, 0.58]; β_*F*_ = 1.30 [0.57, 2.01]; and β_*P*_ = −1.24 [−2.54, 0.12]. When focusing on the results for all parameters, in the facial expression feedback condition, the participants made decisions regarding IGT based on the win frequency more than in the symbolic condition.

To compare the feedback conditions more quantitatively, we checked the group difference by examining the posterior distribution of the conditional mean differences. Generally, in classical statistical hypothesis testing, if the 95% credible interval of the parameters does not include zero, it can be inferred that the effect is significant. Accordingly, there were no differences in conditions for all parameters (*A*_*rew*_ [95% CI] = [−0.12, 0.05]; *A*_*pun*_ [95% CI] = [−0.04, 0.01]; *K* [95% CI] = [−0.34, 0.07]; β_*F*_ [95% CI] = [−0.22, 1.63]; and β_*P*_ [95% CI] = [−2.85, 0.47]).

**FIGURE 3:**
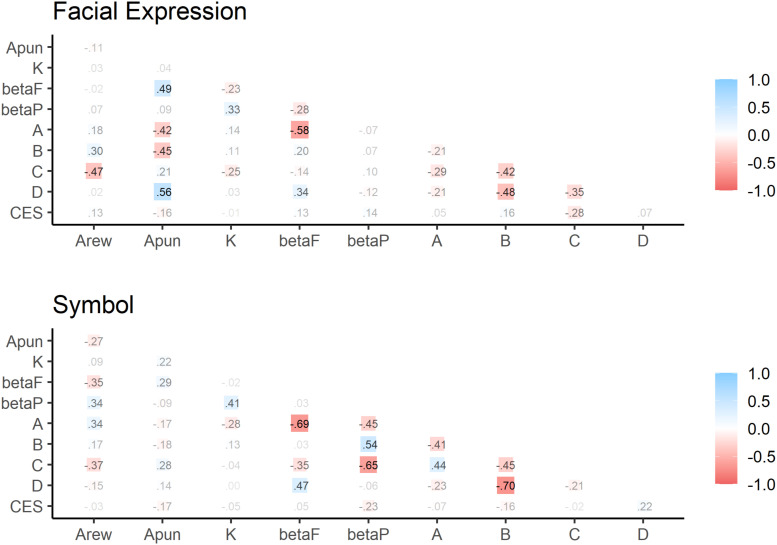
Correlation matrix for Iowa Gambling Task performances, feedback condition, and individual differences. Note: A = the frequency of Deck A choice, B = the frequency of Deck B choice, C = the frequency of Deck C choice, and D = the frequency of Deck D choice. CES = the average scores of all items for the Center for Epidemiological Studies Depression Scale.

Figure 3 shows the correlation matrix for each feedback condition. IGT performance, including the computational parameters, was not significantly correlated with depressive tendency in both conditions (facial expression feedback: *r*s < | 0.28|, *p*s > 0.15 and symbolic feedback: *r*s < | 0.23|, *p*s > 0.25).

## Discussion

This study investigated the communicative effects of facial expressions in learning patterns over time, namely whether facial expressions affect performance in the IGT. As Figure 2 indicates, the learning rate for the case of facial expression feedback was slow. This result was not consistent with the hypothesis that feedback of facial expressions contributes to the credibility of monetary feedback in the IGT and promotes learning. We also found no significant correlations between IGT performance and feedback condition.

According to the theory of affective pragmatics (Scarantino, 2019), facial expressions carry natural information about an emotion, and smiles or frowns and happiness or anger are statistically and probabilistically correlated. Thus, facial expressions do not necessarily indicate a unified meaning. For instance, a smile can be considered as a rewarding smile as well as a dominant smile (Martin et al., 2021), a smile of pain (Kunz et al., 2013), or a distress smile (Singh and Manjaly, 2021). Conversely, ∘ as a positive or correct feedback can be expected to be relatively definitive than a smile, and the relationship it shows between the meaning and the form can be interpreted as being more certain than that in the case of facial expression. Therefore, the effect of feedback on learning was stronger in the symbolic feedback than in the facial expression condition, which caused the difference observed in this study. This result also corresponds with that of Lin et al. (2012). The facial expression may be ambiguous and thus less able to enhance monetary feedback in the learning task.

Moreover, we found that our data could be fitted into the ORL model as normal IGT data (Haines et al., 2018); however, there was no difference between the two conditions in terms of deck frequency or the computational parameters derived from the ORL model. One of the reasons for the current result is that IGT generally uses monetary feedback. Wang et al. (2018) found that emotional expressions influence the behavior of the observer when candy is used as feedback in an economic game, but when money is targeted, those effects disappear. Monetary feedback and a similar framework may make participants more self-centered and less sensitive to social information, such as facial expressions. Thus, future study is necessary to deepen the understanding of communication effects through facial expressions using an alternative reward to money, such as candy.

As is indicated in Figure 2, there is difference in advantageous deck selection between the first 20 and the last 20 trials, but the learning rate was not as good as that found in previous studies (e.g., Drost et al., 2014). It should be noted, however, that Steingroever et al. (2013) used data from many experiments and found that healthy participants might not prefer decks with infrequent losses, which is inconsistent with previous findings using the IGT (Bechara et al., 1994), there might be several reasons why the learning rate is not high in this study. For example, participants in this study may not have been incentivized to learn because they had no actual compensation or rewards. Although there were instructions to assume that the money in the task was real money, it should be acknowledged that the participants’ learning performance would be associated with their real reward.

Further, it can be assumed that depressive symptoms are involved in IGT performance. Must et al. (2013) indicated that depressed persons tended to behave in a more self-focused way, resulting in impaired social decision-making. Case and Olino (2020) found that participants who had high depressive symptoms showed selection of the advantageous decks. However, this study did not support that result, as shown in Figure 3. It is necessary to continue to investigate the IGT by examining the other individual differences. Furthermore, it is possible that the scale responses given after the IGT task might have an effect of the task performance on the subsequent scale response. Therefore, it is recommended that future studies apply counterbalancing for the order of tasks and scales.

This study has provided new evidence for the communicative effect of facial expressions in learning patterns over time, but it had several limitations. The first limitation was the number and nature of the participants. Although the *post-hoc* sensitivity analysis showed adequate power, observed power calculations are not good strategy as Green and MacLeod (2016) suggest. Future studies should employ a large sample size using the strict power simulation. Additionally, because the participants in the study were only undergraduate students and their socio-economic parameters were not measured, it is unclear how far generalization to other groups is appropriate.

The second limitation is that no instruction, on both facial expression and symbolic feedbacks, was provided in this study. Therefore, it is possible that these additional feedbacks may have simply divided the participants’ attention. In fact, a comparison of the differences in the number of advantageous deck choices between the last 20 and first 20 trials for the open data (*N* = 504; Steingroever et al., 2015) and current data shows that open data are more successful in learning (open data: Mean = 3.55, SD = 6.05; this paper: Mean = 1.79, SD = 4.59). Consequently, future studies should make instruction about facial expression feedbacks more explicit. For example, they should present the sequence of both monetary feedbacks and facilitation feedbacks, such as facial expressions, in a sequential manner to not distract attention by the simultaneous presentation of both money and facial expression. For the improvement of future research and open science, the program code has been made public online (see text footnote 1).

Finally, this study used avatar facial expressions, but a previous study showed that processes underlying the perception of virtual versus real emotional faces might differ (Philip et al., 2018). Therefore, evidence using realistic facial expressions should also be obtained. Moreover, there was no quantitative evaluation of how the facial expressions applied in the current study were perceived by the participants. The authenticity of facial expressions has been found to vary depending on the morphology of facial movements (Ambadar et al., 2009; Perusquía-Hernández et al., 2019). The current study created expressions depending on FaceGen. Therefore, future studies should investigate how facial expressions unfold as feedback of learning over the time.

In the use of additional feedback of facial expressions on the IGT, the learning rate was slow around the middle of learning, relative to symbolic feedback. However, no other significant differences were seen in this study in relation to the parameters of computational models or to depressive symptoms as measured by the questionnaires. Taking a close look at an experimental design, such as attention control in which facial movements compose facial stimuli, can enrich future knowledge for communicative effects of facial expressions.

## Data Availability Statement

The datasets presented in this study can be found in online repositories. The names of the repository/repositories and accession number(s) can be found below: https://osf.io/utgeh/?view_only=3b4ebfc226514e438fa843deb6b004b9.

## Ethics Statement

The studies involving human participants were reviewed and approved by The Ethical Committee of the Graduate School of Education, Hiroshima University. The patients/participants provided their written informed consent to participate in this study. Written informed consent was obtained from the individual(s) for the publication of any potentially identifiable images or data included in this article.

## Author Contributions

The author confirms being the sole contributor of this work and has approved it for publication.

## Conflict of Interest

The authors declare that the research was conducted in the absence of any commercial or financial relationships that could be construed as a potential conflict of interest.

## Publisher’s Note

All claims expressed in this article are solely those of the authors and do not necessarily represent those of their affiliated organizations, or those of the publisher, the editors and the reviewers. Any product that may be evaluated in this article, or claim that may be made by its manufacturer, is not guaranteed or endorsed by the publisher.

## Footnotes

1
https://osf.io/utgeh/?view_only=3b4ebfc226514e438fa843deb6b004b9

2
https://github.com/CCS-Lab/hBayesDM/tree/develop/commons/stan_files
